# Synchronous plexiform neurofibroma in the arytenoids and neurofibroma in the parapharynx in a patient with non-neurofibromatosis: a case report

**DOI:** 10.1186/1752-1947-7-15

**Published:** 2013-01-10

**Authors:** Hee Young Son, Hyun Seok Shim, Jin Pyeong Kim, Seung Hoon Woo

**Affiliations:** 1 Department of Otolaryngology, Gyeongsang National University, Jinju, Korea; 2 Department of Otolaryngology, Thyroid/Head and Neck Cancer Center of The Dongnam Institute of Radiological & Medical Sciences (DIRAMS), Pusan, Korea; 3 Institute of Health Sciences, Gyeongsang National University, Jinju, Korea

**Keywords:** Laryngeal neurofibroma, Laryngeal tumor, Plexiform neurofibroma

## Abstract

**Introduction:**

Plexiform neurofibroma of the larynx is a rare disease. In this report, we present a plexiform neurofibroma in the arytenoids and neurofibroma in the parapharynx detected coincidently.

**Case presentation:**

A 56-year-old Asian woman presented with respiratory distress and episodes of apnea at night. A solitary mass from the left arytenoids was found to be nearly obstructing the airway and causing the sleep apnea. There was also a parapharynx mass protruding into the pharynx. The parapharynx tumor was removed with the lateral incision approach, and the arytenoid tumor was removed with a transoral carbon dioxide laser. The pathologic diagnosis was plexiform neurofibroma for the arytenoid mass and neurofibroma for the parapharynx mass.

**Conclusion:**

We have reported an extremely rare case of plexiform neurofibroma in the arytenoids and neurofibroma in the parapharynx. This entity may be considered in the differential diagnosis of all laryngeal and parapharynx masses.

## Introduction

Plexiform neurofibroma, also known as von Recklinghausen disease (VRD), affects Schwann cells. The tissues that grow into tumors may be benign and unencapsulated in an neurogenic tumor. Plexiform neurofibroma is a non-metastatic and locally invasive tumor that can occur on the skin or along the peripheral nerves [1-4].

Plexiform neurofibroma is an unpleasant tumor due to its slow but relentless growth along the nerves in a poorly circumscribed fashion, restricting the possibility of radical surgical resection without postoperative morbidity. Sarcomatous degeneration occurs in 5% of neurofibromas [5]. Large tumors in the neck are usually of the plexiform type and may involve cranial nerves VIII to XII as well as the brachial plexus and sympathetic chain [6].

We present a case of plexiform neurofibroma in the arytenoids and neurofibroma in the parapharynx detected coincidently.

## Case presentation

**Figure 1:**
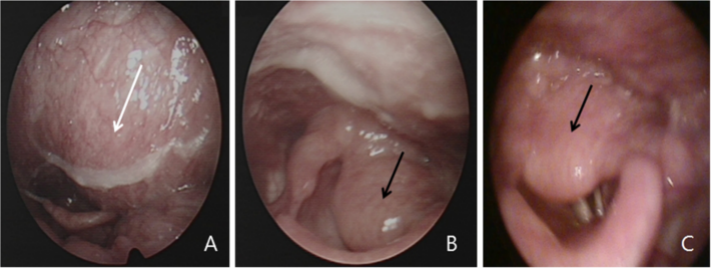
A) Laryngoscopic examination shows a protruding parapharynx mass (white arrow), B) a left arytenoid mass (black arrow), C) the mass is covering the airway (black arrow).

A 56-year-old Asian woman presented with respiratory distress and episodes of apnea at night. During the laryngoscopy, a 1.5×2.0cm tumor was identified in the area of the left arytenoids. The laryngeal inlet was hidden beneath the protruding tumor, but the upper esophageal sphincter was free from the tumor. The upper parapharynx wall of the left vocal cord was protruding toward the pharyngeal cavity (Figure 1). The left vocal cord was fixed. The patient had no previous medical or family history except for hypertension. No enlarged lymph nodes were found in the neck during palpation. The remainder of the physical examination, which included ophthalmologic and dermatologic tests, was also negative. 

**Figure 2:**
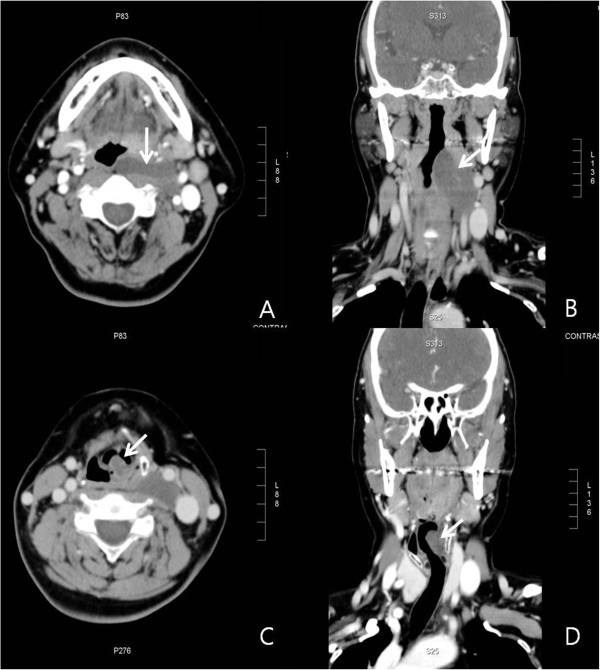
Computed tomography with contrast shows a well-defined homogenous mass lesion involving the parapharyngeal space (white arrows) (A, B) and left arytenoids (white arrows) (C, D).

Computed tomography (CT) revealed a non-enhancing mass arising from the left side of the arytenoid cartilage and from the parapharynx and extending to the areas of the carotid artery (Figure 2). On closer examination of the axial view from the CT image, we were able to observe a mass in the arytenoid cartilage and a mass in the parapharynx existing separately without any linkage at the second cervical section. We suspected a neurogenic tumor originating from the left vagus nerve and superior laryngeal nerve based on the results. Therefore, we planned to remove the mass in the parapharynx with the lateral pharyngotomy approach, in order to excise the mass both in the neck and larynx at the same time, and then advance toward the larynx to remove the mass in the arytenoids. Using the lateral pharyngotomy approach, we found that the cervical mass was 3.5×5.0cm in size with a yellowish color and an ovoid shape. The mass was adherent to the surrounding tissue, resulting in a difficult excision that required removal of tissue surrounding the tumor as well as the tumor itself. We were able to determine that it was a neurogenic tumor due to its linkage to nerves in both the upward and downward directions.

We then examined the mass closely, but it did not connect to the larynx. Thus, we used the lateral pharyngotomy approach for the removal of the parapharynx tumor, and removed the arytenoid tumor using a carbon dioxide (CO_2_) laser. The size of the larynx tumor was 2.0×1.3cm and it enclosed the arytenoids.

**Figure 3:**
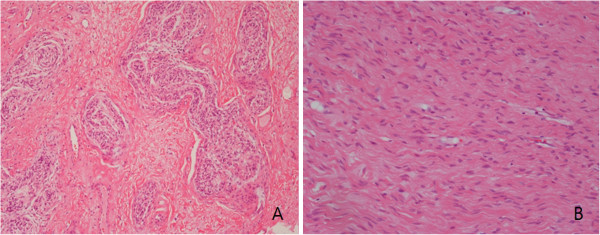
**A) Microscopic findings of the plexiform neurofibroma. **The tumor comprises a tortuous mass of expanded nerve branches (hematoxylin and eosin stain, original magnification ×40). **B) Microscopic findings of the neurofibroma.** Thin spindle cells were associated with thin, wavy collagen bundles. The cells and collagen bundles were loosely spaced in a clear or mucinous matrix (hematoxylin and eosin stain ×200).

The histological stain was positive for S-100, and an examination revealed soft tissue with multiple nerve fascicles, some of which were enlarged with edematous endoneurium with intermixed collagen and bipolar cells consistent with a diagnosis of plexiform neurofibroma; however, the arytenoid mass was confirmed as a simple neurofibroma (Figure 3).

After the pathologic examination, we suspected VRD. In consequence, a close examination was performed but no specific findings were present. The patient complained of a voice disorder postoperatively, and left vocal cord palsy was present on physical examination. We waited and monitored the situation, but the patient showed no improvement; therefore, we conducted an injection laryngoplasty postoperatively, after which the vocal disorder and airway aspiration improved.

**Figure 4:**
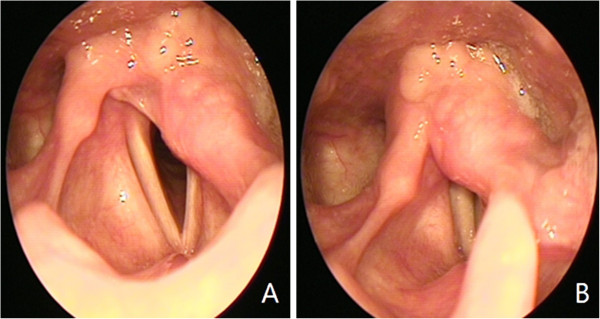
Postoperative laryngoscopic examination shows a fixed left vocal cord (A) and decreased arytenoid mass (B).

Two months later, a direct laryngoscopy showed no tumor growth. To date, two years postoperatively, the patient has had neither respiratory nor feeding difficulties, and her voice is nearly normal (Figure 4).

## Discussion

Among benign tumors, those originating from peripheral nerve tissues are divided into schwannomas and neurofibromas. Plexiform neurofibroma is a benign tumor caused by an abnormal proliferation of peripheral nerves such as Schwann cells, neurons, fibroblastic cells, and peripheral nerve cells, among others [1-4].

Neurofibroma is divided into two groups: plexiform neurofibroma and nonplexiform neurofibroma. Nonplexiform neurofibroma is more common, and because it is easily distinguishable from the surrounding tissues it is possible to fully excise it during surgery.

Nonplexiform neurofibroma displays particular pathologic findings, such as the formation of spindle cells and an abundance of collagen around the center of the tumor. Plexiform neurofibroma has a similar structure to nonplexiform neurofibroma; however, it spreads widely around the cranial nerves, the skin, and the peripheral nerves of the intestinal organs. In addition, it is unencapsulated, more invasive, and indistinguishable from the surrounding tissues, resulting in difficult excision. As a result, it is likely to recur after surgery [7].

Clinical symptoms of neurofibroma vary according to the size and location of the tumors. The involvement of the larynx can lead to dysphagia and difficulty with respiration. Disorders such as Horner’s syndrome and vocal palsy are seen in cases that develop in the cervical region. Growth of the tumor is slow, and mild symptoms sometimes lead to its discovery, as was the case here, even though the tumor was small.

In this case, different neurogenic tumors formed simultaneously in the main trunk of the vagus nerve and in its superior laryngeal nerve branch. We discovered that the tumors were not linked to each other during the initial surgery; therefore, we opted to proceed with an appropriate surgery for each tumor instead of removing the entire tumor all at once along with the vagus nerve. This case is very rare and is believed to be the first of its kind [2].

CT and magnetic resonance imaging (MRI) scans are very useful in diagnosing neurofibroma. In this case, CT demonstrated a tumor surrounding an area of low attenuation. There was no significant enhancement [3].

S-100 immunohistochemistry is helpful in differentiating between spindle cell tumors and neurofibroma. In this case, the tumor in the parapharynx was diagnosed as a plexiform neurofibroma and the tumor in the arytenoid region was diagnosed as a nonplexiform neurofibroma from the biopsy results. The results confirmed that the two tumors had different patterns.

The treatment for neurofibroma can vary depending on clinical symptoms, size and location of the tumor, and subtype, and complete excision should be performed in order to prevent recurrence.

A small-sized nonplexiform neurofibroma located in the supraglottis can be removed with a suspension laryngoscope, and a large-sized tumor in the supraglottis, as well as all tumors in the subglottis, should generally be removed using an external approach. External approaches such as lateral pharyngotomy, laryngotomy, or lateral thyrotomy can be used for excision [8-10].

In this case, the tumors formed in both the vagus nerve and its superior laryngeal nerve branch; therefore, vocal movement was affected postoperatively. With use of injection laryngoplasty for vocal cord medialization, which has been recently upgraded, vocal disorders and aspiration can be prevented.

Rao *et al*. reported monitoring a 4-year-old girl who had plexiform neurofibroma surrounded by vital organs from the thorax to the abdomen, because the tumors do not compress the surrounding tissues [11]. However, when considering the formation patterns of plexiform neurofibroma, there is controversy over a report that states it is crucial to remove all of the tumor at all costs (leaving the patient with large neurological deficits) due to its growth pattern. There is a tendency for the tumor to recur from microinvasion of the involved nerves, subsequently affecting the patient’s quality of life [12].

Neurofibroma is a benign disease, but people with the disease have a higher risk of developing a variety of other tumors. It has been reported that from 5% to 13% of spindle nonplexiform neurofibromas are converted to malignant tumors [13]. These types of tumors will greatly affect the prognosis; therefore, it is necessary to closely monitor patients with these benign tumors to prevent future complications.

In this case, plexiform neurofibroma formed in the vagus nerve and nonplexiform neurofibroma formed in the superior laryngeal nerve, which is a peripheral branch of the vagus nerve. We removed the tumors using an appropriate approach for each tumor.

## Conclusion

We present a case of plexiform neurofibroma in the arytenoid and neurofibroma in the parapharynx detected coincidently. We removed the masses in the parapharynx and arytenoids, and the patient has had no specific findings to date. This entity may be considered in the differential diagnosis of all laryngeal and parapharynx masses.

## Consent

Written informed consent was obtained from the patient for publication of this case report and any accompanying images. A copy of the written consent is available for review by the Editor-in-Chief of this journal.

## Competing interests

The authors declare that they have no competing interests.

## Authors’ contributions

HSS examined our patient, analyzed and interpreted investigative data and wrote the manuscript. HYS examined and analyzed the investigative data. JPK performed a literature review and contributed to writing the manuscript. SHW made critical revisions and contributed to the manuscript writing. All authors read and approved the final manuscript.
